# Luteolin improves the systolic and diastolic functions of the thoracic aortic vessels in type 2 diabetic rats through the Kv7.1 channel

**DOI:** 10.3389/fphar.2025.1651847

**Published:** 2025-09-16

**Authors:** Weiping Li, Zerong Yang, Xingru Wang, Pengmei Guo, Li Wu, Yanru Wang, Yue Lyu, Xiaojia Xu, Haijie Ji

**Affiliations:** ^1^ Department of Pharmacology, Fenyang College, Shanxi Medical University, Fenyang, China; ^2^ Cultivation Key Laboratory of Metabolic Cardiovascular Diseases Research, Fenyang College, Shanxi Medical University, Fenyang, China; ^3^ Innovative Institute of Chinese Medicine and Pharmacy, Academy for Interdiscipline, Chengdu University of Traditional Chinese Medicine, Chengdu, China; ^4^ Shanxi Key Laboratory of Chinese Medicine Encephalopathy, Shanxi University of Chinese Medicine, Jinzhong, China; ^5^ Shanxi Province Academy of Traditional Chinese Medicine, Taiyuan, China

**Keywords:** luteolin, type 2 diabetic rats, thoracic aorta, vasomotion, Kv7.1

## Abstract

**Objectives:**

This study aimed to evaluate the therapeutic effects of luteolin (Lut) on vascular dysfunction in type 2 diabetic rats and explore its underlying mechanisms, particularly its regulation of the myogenic response in thoracic aortic vessels via the Kv7.1 (KCNQ1) channel.

**Methods:**

Type 2 diabetes mellitus (T2DM) was induced in rats via high-fat/high-glucose diet combined with intraperitoneal streptozotocin (30 mg/kg). Animals were assigned to four groups: normal control (NC), NC + Lut (80 mg/kg), diabetic (DM), and DM + Lut. Fasting blood glucose, body weight, lipid profile, and blood pressure were monitored. Myogenic response of the thoracic aorta was assessed using vascular ring tension assays. Expression of KCNQ1 was evaluated via qRT-PCR. *In vitro*, A7r5 cells were cultured under normal (5.5 mM) or high glucose (30 mM) conditions, with or without the addition of chromanol 293B (Kv7.1 inhibitor) or PDBu (PKC agonist), the effects of Lut on the expression of KCNQ1 and Kv7.1 were observed by qRT-PCR and cellular immunofluorescence assay.

**Results:**

Luteolin significantly reduced fasting blood glucose, lowered blood pressure, and improved lipid parameters in diabetic rats. It attenuated the enhanced vasoconstriction and impaired vasodilation observed in DM rats. KCNQ1 expression was downregulated in DM rats but restored by Lut treatment. In A7r5 cells, Lut increased KCNQ1 and Kv7.1 expression, which was inhibited by high glucose or PKC activation.

**Conclusion:**

Luteolin improves vascular tone and function in diabetic rats by restoring Kv7.1/KCNQ1 expression, possibly through inhibition of PKC signaling. These findings highlight Kv7.1 as a potential therapeutic target for diabetic vascular complications.

## 1 Introduction

Diabetes causes a variety of vascular complications in the body, causing irreversible damage to blood vessels, such as microvascular complications and large and medium vascular complications (Guo et al., 2020), and cardiovascular disease is the main cause of death from diabetes. Basic medical research has shown that diabetes changes the myogenic response of blood vessels to many vasoactive substances in the body, and long-term diabetes will increase the incidence of hypertension, while many diabetic patients with hypertension show a kind of refractory hypertension (Solini et al., 2014; Takahashi et al., 2016), which increases the difficulty of clinical treatment of the disease. Patients with borderline impaired glucose tolerance or type 2 diabetes have more severe atherosclerosis than those with normal glucose tolerance (Salomaa et al., 1995). Therefore, the study of drugs that can both lower blood glucose and improve vascular myogenic response disorders will benefit diabetic hypertensive patients. However, the drugs that can reduce blood sugar and protect vascular damage are very limited in clinical practice, and actively searching for new therapeutic drugs has become a key research content in the field of diabetes and hypertension treatment. Although new hypoglycemic drugs have been emerging in recent years, they do not directly improve the myogenic response of blood vessels. For example, the Sodium-glucose cotransporter 2 (SGLT2) inhibitor dagliprazin and glucagon-like peptide-1 (GLP-1) receptor agonist semaglutide can only protect the cardiovascular system by improving blood glucose and lipid metabolism (Sarafidis and Tsapas, 2016; Ma et al., 2021).

Luteolin is a kind of flavonoid, widely found in various natural plants and natural foods. Studies have shown that luteolin is beneficial to cardiovascular disease (Huang et al., 2023), including dilating blood vessels, reducing blood lipids, inhibiting smooth muscle cell proliferation, inhibiting angiogenesis, improving myocardial fibrosis, and anti-atherosclerosis. If luteolin can improve the myogenic response disorder of blood vessels in diabetes, it will delay the damage of blood vessels in diabetes and bring good news to diabetic hypertensive patients. The purpose of this study is to determine the therapeutic effect of luteolin on diabetic rats and whether it can improve the vascular myogenic response disorder of thoracic aorta, so as to provide data support for its potential treatment of diabetic hypertension.

## 2 Results

### 2.1 Body weight, blood glucose, arterial pressure and biochemical indexes were detected in each group

**FIGURE 1:**
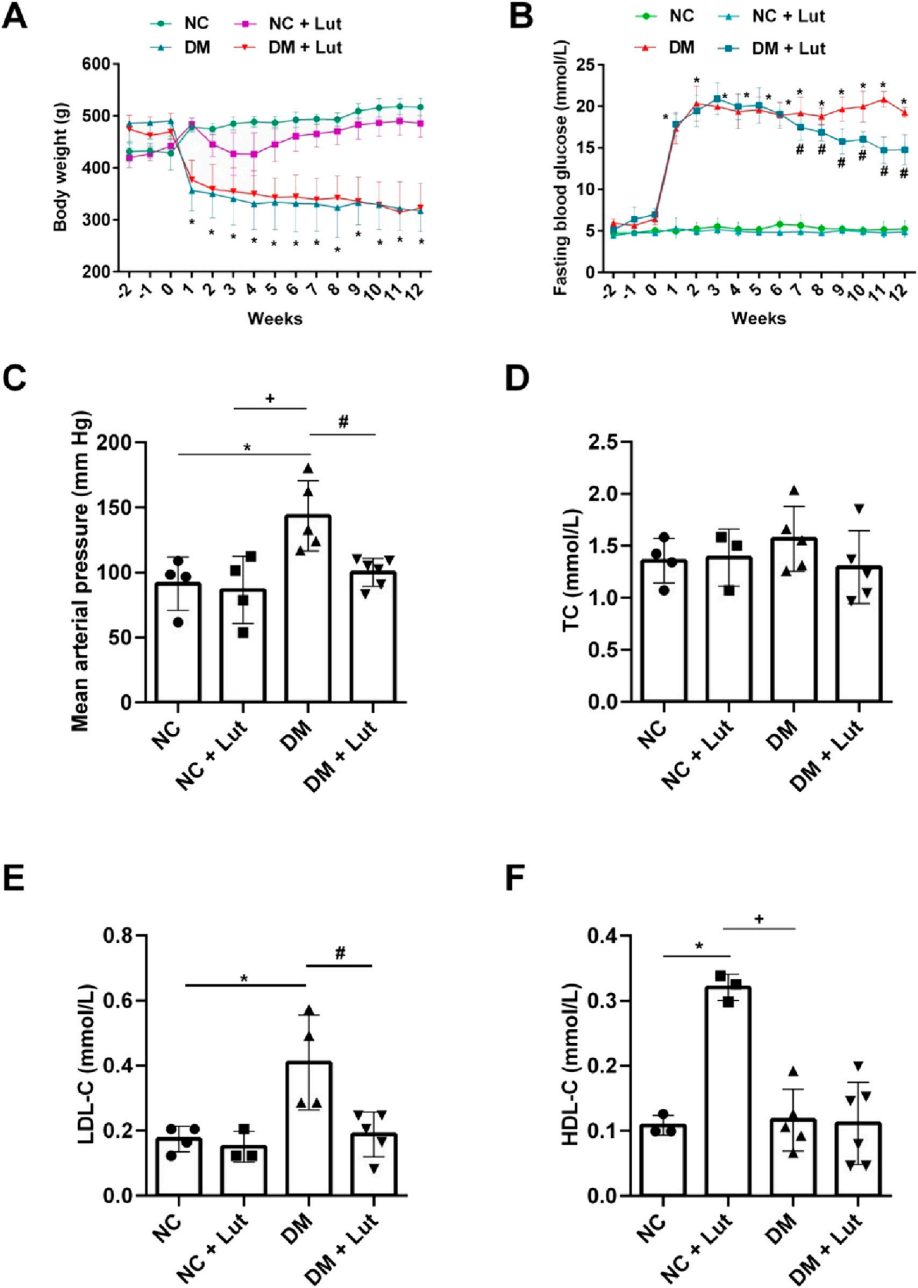
Body weight, blood glucose and biochemical tests were performed in each group. Note: **(A)** The body weight value of rats in each group. **(B)** The fasting blood glucose value of each group. **(C)** Mean arterial pressure in each group. **(D)** The value of total cholesterol (TC) in each group. **(E)** The low-density lipoprotein cholesterol (LDL-C) value of rats in each group. **(F)** The high-density lipoprotein cholesterol (HDL-C) value of rats in each group. One-way ANOVA was used to analyze the data. *P < 0.05 vs. NC (normal control), ^+^ *P* < 0.05 vs. NC + Lut, ^#^ *P* < 0.05 vs. DM.

As shown in Figure 1A, there was no significant difference in body weight between the normal control group (NC) and the NC + Lut group from the 1st to 12th week (*P* > 0.05), the body weight of diabetes mellitus (DM) group was significantly decreased than that of NC (*P* < 0.05), but there was no significant difference between the DM + Lut group and DM group about the body weight (*P* > 0.05). Figure 1B shows the changes of blood glucose over time. From the 1st to 12th week, there was no significant difference in blood glucose between NC + Lut group and NC (*P* > 0.05), the blood glucose of DM group was significantly higher than that of NC (*P* < 0.05), but the blood glucose of DM + Lut group was significantly decreased than that of DM group at the 7th to 12th week (*P* < 0.05). At the end of experiment, the mean arterial pressure (MAP) of rats in each group was detected. The MAP of rats in NC + Lut group had no significant change compared with the NC group (*P* > 0.05, Figure 1C), but the MAP was significantly increased in DM group compared with the NC group (*P* < 0.05). The MAP of DM + Lut group was significantly decreased than that of DM group (*P* < 0.05). The MAP in DM + Lut group was higher than that in control group, but there was no significant difference (*P* > 0.05).

After 12 weeks of feeding, serum was collected to detect biochemical indexes, there was no significant difference in total cholesterol (TC) among NC, NC + Lut, DM and DM + Lut groups (*P* > 0.05, Figure 1D). There was no significant difference in LDL-C between the NC + Lut group and the NC group (*P* > 0.05), but the LDL-C of DM group was significantly higher than that in NC group (*P* < 0.05), and LDL-C in the (DM + Lut) group was significantly decreased than that in DM group (*P* < 0.05, Figure 1E). HDL-C was significantly higher in (NC + Lut) group than in NC (*P* < 0.05, Figure 1F). DM group was significantly decreased than (NC + Lut) group (*P* < 0.05), but there was no significant difference between (DM + Lut) group and DM group about HDL-C (*P* > 0.05).

### 2.2 The thoracic aorta vasoconstriction were measured

**FIGURE 2:**
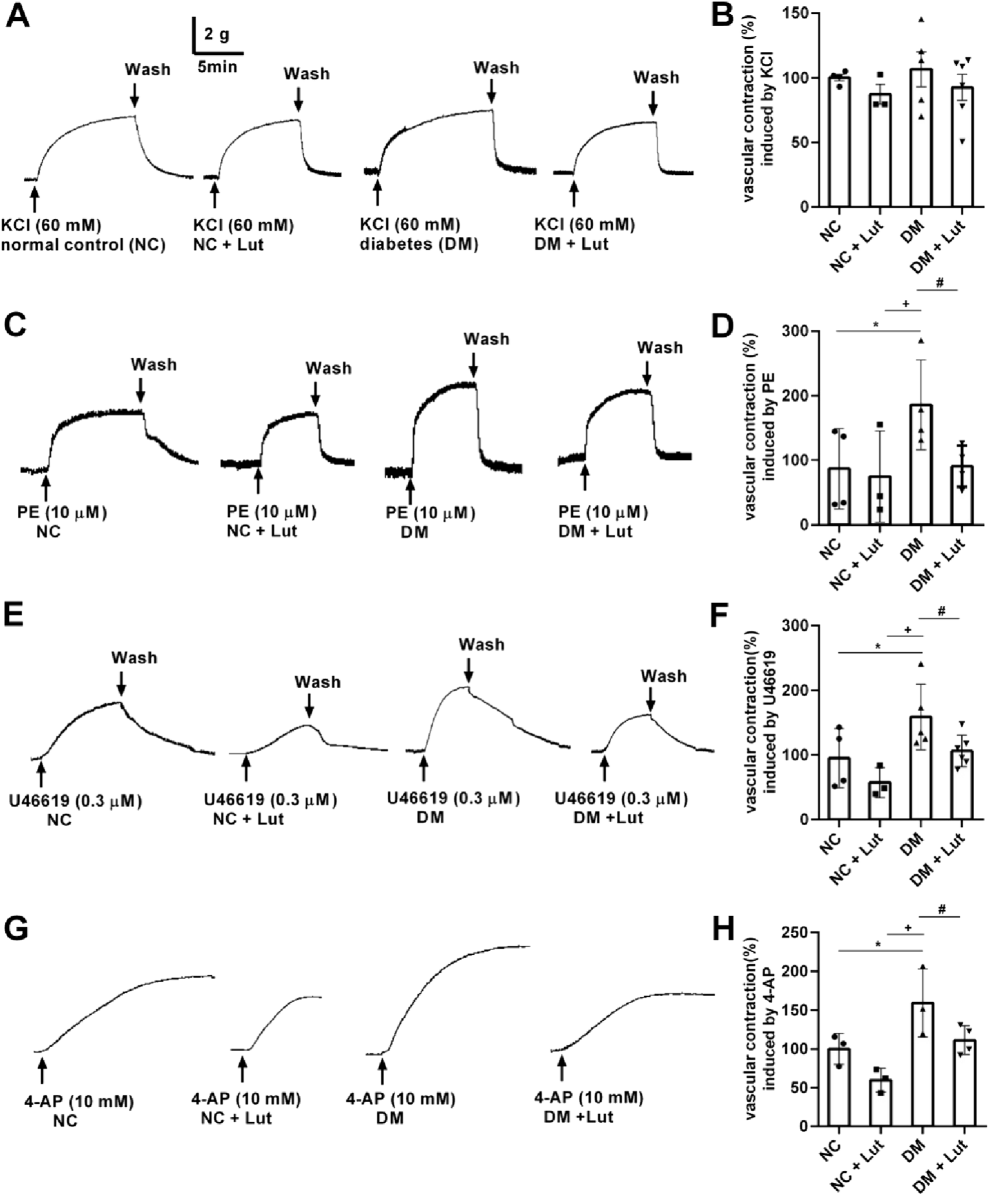
RTAs contractions induced by KCl, PE, U46619 or 4-AP. Note: The vasoconstriction responses of isolated RTAs to KCl (2A), PE (2C), U46619 (2E), 4-AP (2G) were recorded in normal control (NC), NC + Lut, diabetes mellitus (DM), or DM + Lut group. **(B,D,F,H)** Pooled data of **(A,C,E,G)**. Vasoconstriction induced by 60 mM KCl, 10 μM PE, 0.3 μM U46619 and 10 mM 4-AP in NC group were taken as 100%, respectively. The results were expressed as means ± SD, n = 3–6. One-way ANOVA was used to analyze the data. *P < 0.05 vs. NC, ^+^ *P* < 0.05 vs. NC + Lut, ^#^ *P* < 0.05 vs. DM.

Figures 2A,B shows that there were no significant differences in vasoconstriction of rat thoracic aorta rings (RTAs) induced by 60 mM KCl among the normal control (NC), NC + Lut, DM, and DM + Lut groups (*P* > 0.05). In Figures 2C,D, compared with NC group or NC + Lut group, RTAs vasoconstriction induced by 10 μM PE was significantly increased in DM group (*P* < 0.05); compared with NC group, the RTAs contraction amplitude in NC + Lut group was decreased but there was no significant difference (*P* > 0.05). Compared with the DM group, the RTAs vasoconstriction caused by 10 μM PE was significantly decreased in DM + Lut group (*P* < 0.05). In Figures 2E,F, RTAs vasoconstriction induced by 0.3 μM U46619 was significantly higher in DM group than in NC group or in NC + Lut group (*P* < 0.05). Compared with NC group, the RTAs contraction amplitude in NC + Lut group was decreased but there was no significant difference (*P* > 0.05). Compared with DM group, RTAs vasoconstriction induced by 0.3 μM U46619 was significantly decreased in DM + Lut group (*P* < 0.05). In Figures 2G,H, compared with NC group or NC + Lut group, RTAs vasoconstriction induced by 10 mM 4-AP was significantly increased in DM group (*P* < 0.05). The amplitude of vasoconstriction in NC + Lut group was lower than that in NC group, but there was no obvious difference (*P* > 0.05). RTAs vasoconstriction induced by 10 mM 4-AP was significantly reduced in the DM + Lut group compared with the DM group (*P* < 0.05).

### 2.3 The thoracic aorta vasorelaxation were measured

**FIGURE 3:**
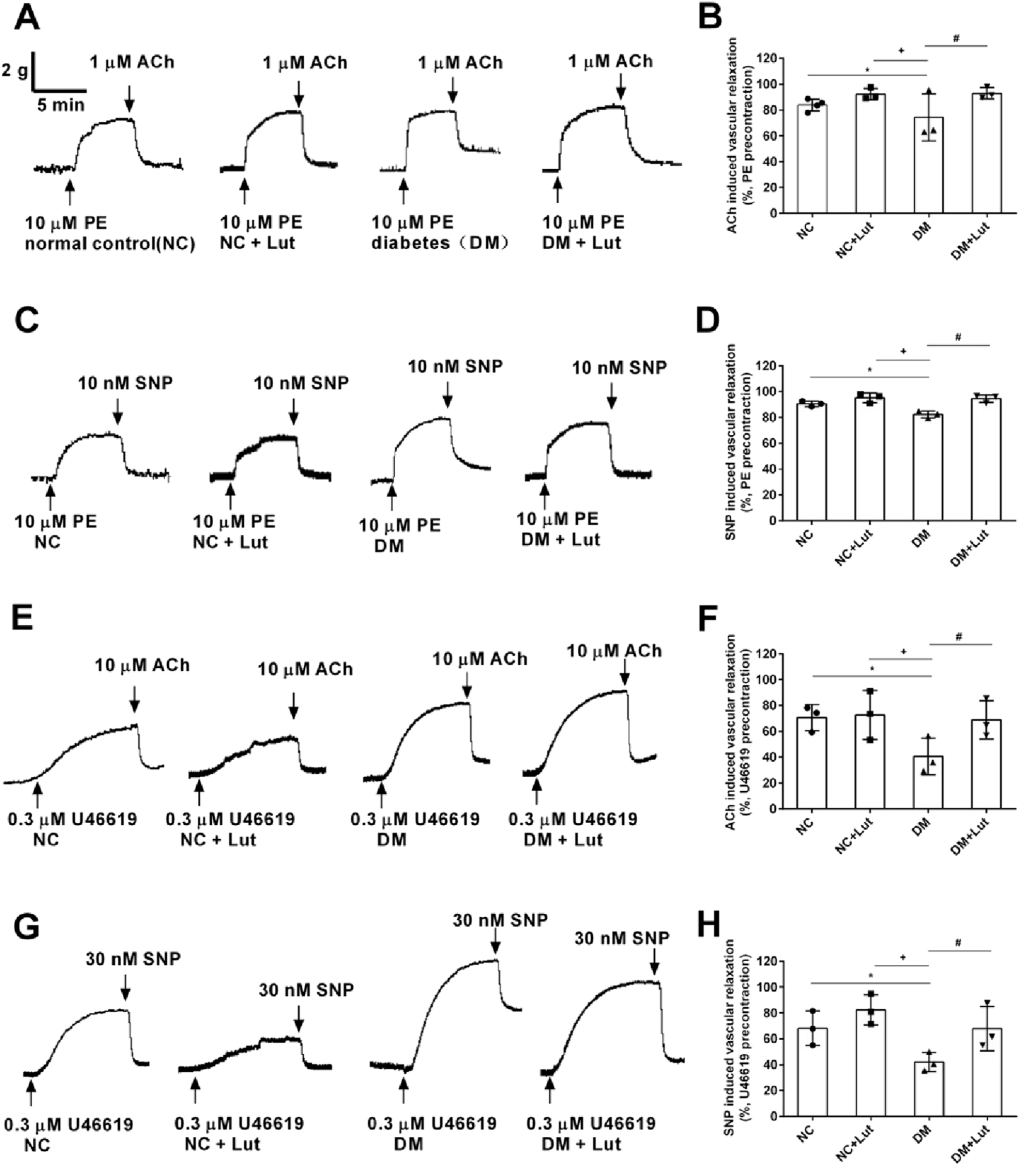
ACh or SNP relaxed rat thoracic aorta rings (RTAs) precontracted with either10 μM PE or 0.3 μM U46619 in each group. Note: **(A,C)** The original traces of ACh induced vasorelaxation **(A)** or SNP induced vasorelaxation **(C)** in RTAs precontracted with 10 μM PE. **(B,D)** Pooled data of **(A,C)**. **(E,G)** The original traces of ACh induced vasorelaxation **(E)** or SNP induced vasorelaxation **(G)** in RTAs precontracted with 0.3 μM U46619. **(F,H)** Pooled data of **(E,G)**. Vasorelaxant responses (means ± SD) were expressed as percentages of the respective precontractions, n = 3. One-way ANOVA was used to analyze the data. *P < 0.05 vs. NC (normal control); ^+^ *P* < 0.05 vs. NC + Lut; ^#^ *P* < 0.05 vs. DM.

Acetylcholine (ACh) was used to dilate the thoracic aortic ring, which was precontracted with 10 μM PE. In Figures 3A,B, the results showed that the diastolic percentage of thoracic aortic ring in NC + Lut group was not significantly different from that in NC group (*P* > 0.05); compared with NC group and NC + Lut group, the diastolic percentage of DM group was significantly decreased (*P* < 0.05); there was no significant difference between DM + Lut group and NC group (*P* > 0.05); but compared with DM group, the diastolic percentage was significantly higher in DM + Lut group (*P* < 0.05). PE was used to precontract the thoracic aortic vascular ring, and SNP was used to dilate the blood vessels. In Figures 3C,D, the results showed that the diastolic percentage of thoracic aortic vascular ring in NC + Lut group was not obviously different from that in NC group (*P* > 0.05); compared with NC group or NC + Lut group, the diastolic percentage of DM group was significantly decreased (*P* < 0.05); there was no significant difference in vasodilation percentage between DM + Lut group and NC group (*P* > 0.05); however, compared with DM group, the diastolic percentage in DM + Lut group was significantly higher (*P* < 0.05).

U46619 was used to precontract the vascular ring of the thoracic aorta. After reaching the plateau, ACh was used to dilate the vessels. In Figures 3E,F, the results showed that there was no significant difference about the diastolic percentage of thoracic aortic vascular ring between the NC + Lut group and the NC group (*P* > 0.05); compared with the NC group or the NC + Lut group, the percentage of vasodilation of thoracic aorta was significantly reduced in DM group (*P* < 0.05); there was no significant difference in the percentage of aortic ring relaxation between DM + Lut group and NC group (*P* > 0.05); however, compared with DM group, the percentage of aortic ring relaxation in DM + Lut group was significantly increased (*P* < 0.05). SNP was used to relax the vascular ring of thoracic aorta, which was precontracted with 0.3 μM U46619. In Figures 3G,H, the results showed that there was no significant difference between the NC + Lut group and the NC group in the diastolic percentage of thoracic aortic ring (*P* > 0.05); compared with NC group or NC + Lut group, the percentage of vasodilation in DM group was significantly decreased (*P* < 0.05); there was no significant difference in the percentage of vascular ring relaxation between DM + Lut group and NC group (*P* > 0.05); however, compared with DM group, the diastolic percentage of vascular ring in DM + Lut group was significantly increased (*P* < 0.05). It is speculated that luteolin can improve the vasodilation function in diabetic rats, including both endothelium-dependent and non-endothelium-dependent diastolic function.

### 2.4 The Kv7 channel is related to vasodilatory function of luteolin

**FIGURE 4:**
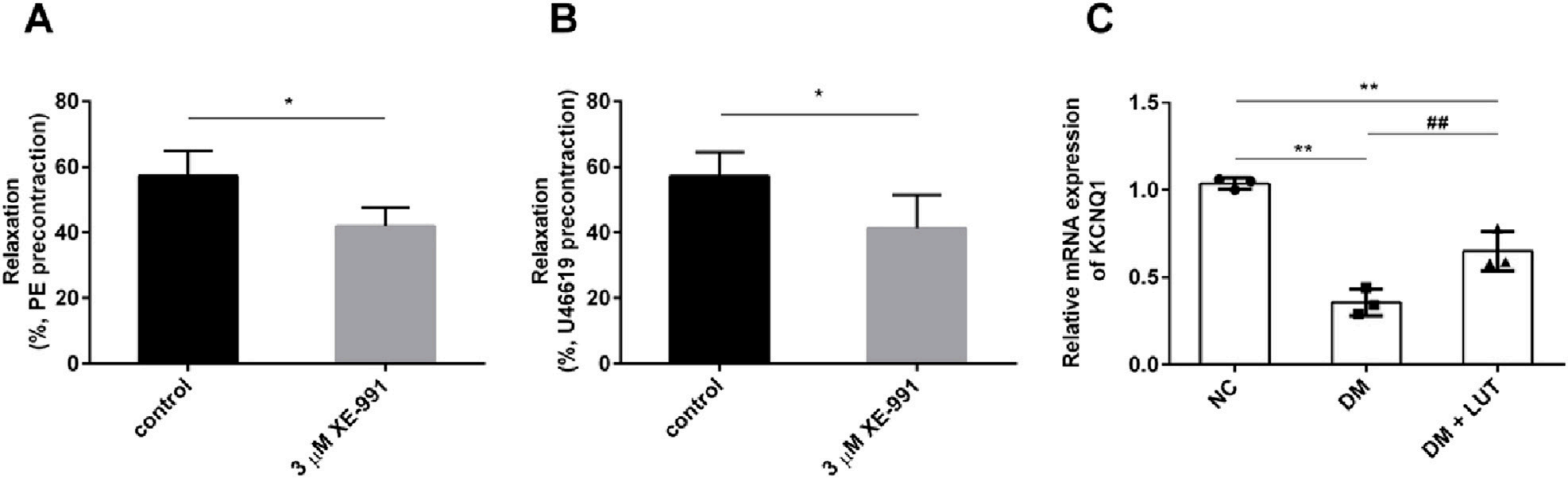
To investigate the effect of XE-991 on the vascular relaxation of thoracic aorta in diabetic rats and the mRNA level of KCNQ1 in thoracic aorta in each group. Note: **(A,B)** The thoracic aortic vascular rings of diabetic rats were pre-constricted with 10 μM PE **(A)** or 0.3 μM U46619 **(B)**. When the constriction reached the plateau, the percentage of vascular dilation by SNP was observed with XE-991 (3 μM XE-991) or without XE-991 (control). XE-991was Kv7 inhibitor. The diastolic percentage is equal to the diastolic amplitude caused by SNP divided by the pre-contraction amplitude of PE or U46619. **(C)** Detect the mRNA level of KCNQ1 in rats of normal control (NC), diabetes mellitus (DM) and DM + Lut groups. n = 3. T-test was used for A and B, one-way ANOVA was used for **(C)**. *P < 0.05 vs. control; **P < 0.01 vs. NC (normal control); ^##^ *P* < 0.01 vs. DM.

As shown in the Figure 4, for the thoracic aorta of rats precontracted with PE (Figure 4A) or U46619 (Figure 4B), the diastolic amplitude of the vascular ring for SNP was significantly reduced when adding 3 μM XE-991 (*P* < 0.05); it is suggested that the vasorelaxation of the thoracic aorta in diabetic rats caused by SNP is related to the Kv7 channel. The mRNA levels of KCNQ1 in each group of rats were further detected in Figure 4C, it was found that the mRNA level of KCNQ1 in diabetic rats was significantly lower than that in the normal control group (NC) (*P* < 0.01); compared with the diabetes mellitus group (DM), the mRNA level of KCNQ1 in the Lut treatment group (DM + Lut) was significantly increased (*P* < 0.01). It is suggested that the dilatory effect of Lut on the thoracic aorta of diabetic rats may be related to the mRNA level of KCNQ1.

### 2.5 Lut enhanced KCNQ1 and Kv7.1 channel proteins in A7r5 cells with normal glucose condition

**FIGURE 5:**
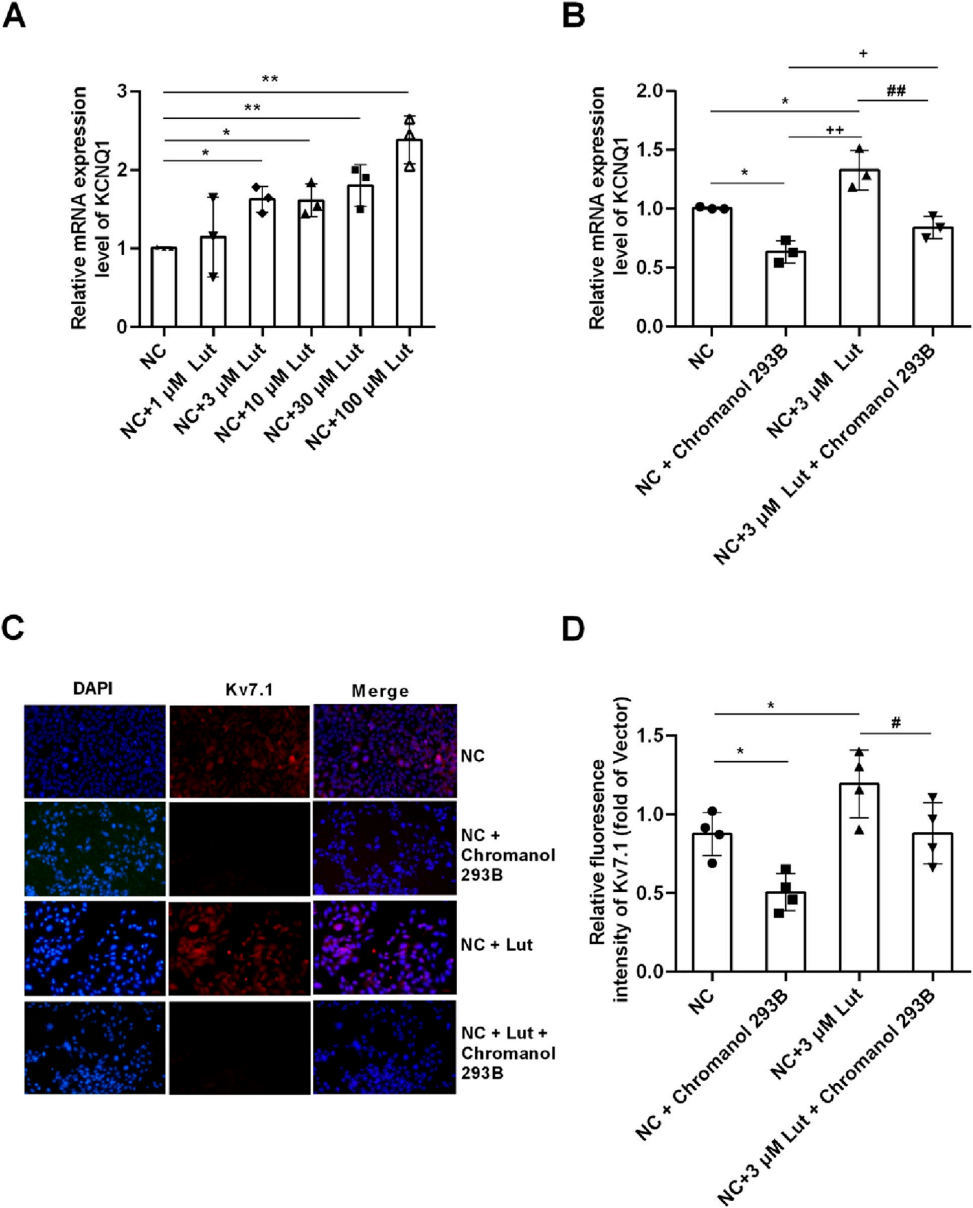
Lut enhanced the expression of KCNQ1 and Kv7.1 proteins in A7r5 cells. Note: **(A)** Lut enhanced the expression of KCNQ1 in a concentration-dependent manner. **(B)** Chromanol 293B (Kv7.1 inhibitor) can significantly inhibit the expression of KCNQ1. **(C)** Lut can enhance the expression of Kv7.1 protein, and Chromanol 293B can reduce the expression of Kv7.1. **(D)** shows the statistical results of **(C)**. n = 3–4. One-way ANOVA was performed for data analysis. *P < 0.05 and **P < 0.01 vs. NC (normal control); ^+^ *P* < 0.05 and ^++^ *P* < 0.01 vs. NC + Chromanol 293B; ^#^ *P* < 0.05 and ^##^ *P* < 0.01 vs. NC + Lut.

The results showed that compared with control group (NC), Lut had no significant effect on KCNQ1 expression at 1 μM (*P* > 0.05), but Lut significantly enhanced KCNQ1 expression at 3 μM, 10 μM, 30 μM or 100 μM (Figure 5A, *P* < 0.05). However, there was no significant difference among 3 μM, 10 μM and 30 μM Lut groups (*P* > 0.05). And there were significant differences between 100 μM Lut group and the 3 μM, 10 μM or 30 μM Lut groups, respectively (*P* < 0.05). Lut 3 μM was selected for subsequent experiments, considering the possible cytotoxicity of Lut at 100 μM. Chromanol 293B is a Kv7.1 inhibitor, as shown in Figure 5B, compared with NC group, NC + Chromanol 293B group can significantly inhibit the expression of KCNQ1 (*P* < 0.05); NC + 3 μM Lut group can significantly enhance the expression of KCNQ1 compared with NC group (*P* < 0.05); NC + 3 μM Lut + Chromanol 293B group significantly weakened the effect of 3 μM Lut on enhancing KCNQ1 expression compared with NC + 3 μM Lut group (*P* < 0.05). As shown in Figures 5C,D, compared with NC group, NC + Chromanol 293B group could significantly inhibit the Kv7.1 fluorescence intensity (*P* < 0.05); NC + 3 μM Lut group could significantly enhance the fluorescence intensity of Kv7.1 compared with NC group (*P* < 0.05); compared with NC + 3 μM Lut group, NC + 3 μM Lut + Chromanol 293B group significantly reduced the immunofluorescence intensity of Kv7.1 (*P* < 0.05). Therefore, 3 μM Lut can significantly enhance the expression of KCNQ1 and Kv7.1 proteins.

### 2.6 Lut improved the expression of KCNQ1 and Kv7.1 proteins in A7r5 cells with high glucose condition

**FIGURE 6:**
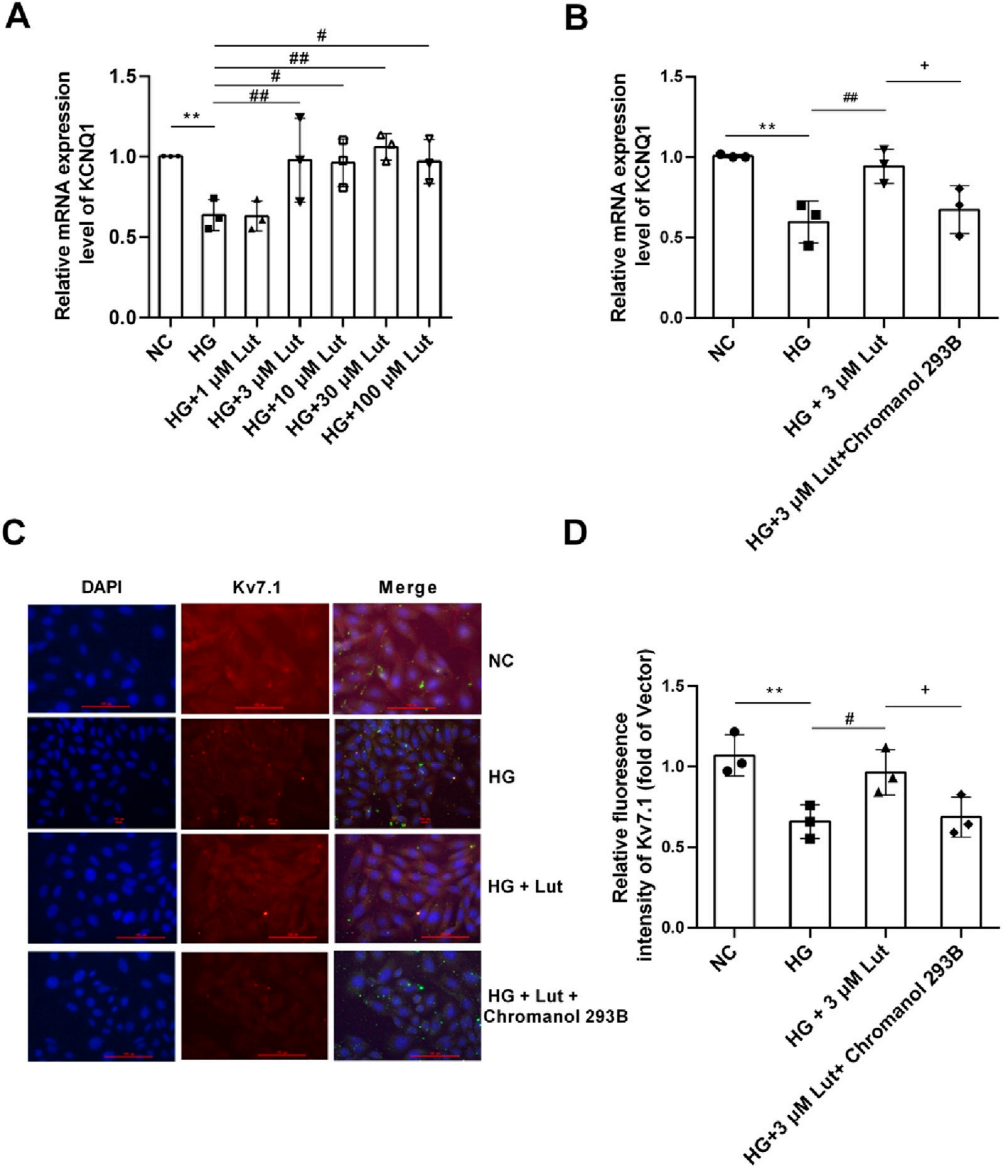
Lut can improve the expression of KCNQ1 and Kv7.1 proteins in A7r5 cells under high glucose condition. Note: **(A)** Expression of KCNQ1 (the gene encoding Kv7.1 protein) were measured in normal control (NC) group, high glucose (HG) group, HG + 1 μM Lut, HG + 3 μM Lut, HG + 10 μM Lut, HG + 30 μM Lut, and HG + 100 μM Lut group. **(B)** The expression of KCNQ1 in NC, HG, HG + 3 μM Lut, HG + 3 μM Lut + Chromanol 293B groups was detected, and Chromanol 293B was an inhibitor of Kv7.1. **(C)** The immunofluorescence image of Kv7.1 in NC, HG, HG + 3 μM Lut, HG + 3 μM Lut + Chromanol 293B groups. Kv7.1 showed red fluorescence with DAPI nucleation. **(D)** is the statistical result graph of **(C)** n = 3. One-way ANOVA was performed for data analysis. **P < 0.01 vs. NC; ^#^ *P* < 0.05 and ^##^ *P* < 0.01 vs. HG; ^+^ *P* < 0.05 vs. HG + 3 μM Lut.

As shown in Figure 6A, compared with normal glucose (NC, 5 mM glucose) group, the expression of KCNQ1 in A7r5 cells in high glucose (HG, 30 mM glucose) group was significantly decreased (*P* < 0.05). There was no significant change in the expression of KCNQ1 between HG + 1 μM Lut group and HG group (*P* > 0.05). But compared with HG group, the expression of KCNQ1 in the HG + (3, 10, 30, 100 μM) Lut group were significantly increased (*P* < 0.01, *P* < 0.05, *P* < 0.01, *P* < 0.05). As shown in Figure 6B, A7r5 cells were cultured and the experiment was divided into four groups. Compared with NC group, the expression of KCNQ1 in HG group was significantly decreased (*P* < 0.01). The expression of KCNQ1 in HG + 3 μM Lut group was significantly higher than that in HG group (*P* < 0.01), but which had no significant difference compared with NC group (*P* > 0.05). Compared with HG + 3 μM Lut group, the expression of KCNQ1 in HG + 3 μM Lut + Chromanol 293B group was significantly decreased (*P* < 0.05), but there was no significant difference compared with HG group (*P* > 0.05). As shown in Figures 6C,D, the protein expression of Kv7.1 was detected by immunofluorescence, and the results showed that the fluorescence intensity of Kv7.1 in the HG group was significantly decreased (*P* < 0.01), while the fluorescence intensity of Kv7.1 in the HG + 3 μM Lut group was significantly enhanced compared with that in the HG group (*P* < 0.05). Compared with HG + 3 μM Lut group, the fluorescence intensity of KCNQ1 in HG + 3 μM Lut + Chromanol 293B group was significantly decreased (*P* < 0.05), but there was no significant difference compared with HG group (*P* > 0.05).

### 2.7 PDBu mediated Lut to improve the expression of KCNQ1 and Kv7.1 proteins in A7r5 cells with normal glucose and high glucose

**FIGURE 7:**
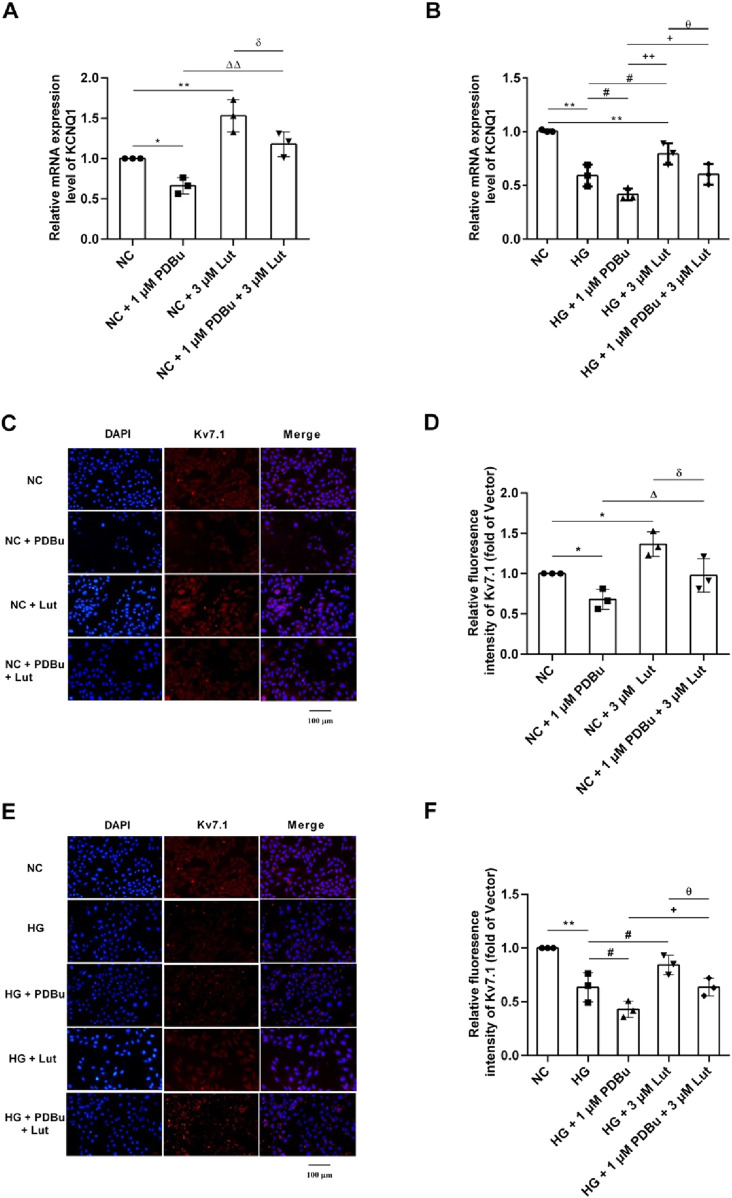
Effect of PDBu (PKC agonist) on the expression of KCNQ1 and Kv7.1 in A7r5 cells enhanced by Lut. Note: **(A)** The effect of 1 μM PDBu on KCNQ1 in A7r5 cells under normal conditions. **(B)** The effect of 1 μM PDBu on KCNQ1 in A7r5 cells under high glucose conditions. **(C)** Effect of PDBu on the expression of Kv7.1 protein in A7r5 cells under normal conditions. **(D)** is the statistical result of **(C)**. **(E)** Effect of PDBu on the expression of Kv7.1 protein in A7r5 cells under high glucose conditions. **(F)** is the statistical result of **(E)** n = 3. One-way ANOVA was performed for data analysis. *P < 0.05 and **P < 0.01 vs. NC; ^#^ *P* < 0.05 vs. HG; ^δ^ *P* < 0.05 vs. NC + 3 μM Lut; ^Δ^ *P* < 0.05 and ^ΔΔ^ *P* < 0.01 vs. NC + 1 μM PDBu; ^+^ *P* < 0.05 and ^++^ *P* < 0.01 vs. HG + 1 μM PDBu; ^θ^ *P* < 0.05 vs. HG + 3 μM Lut.

As shown in Figure 7A, compared with NC (normal control, cell medium was 5 mM Glucose) group, 1 μM PDBu could significantly inhibit the expression of KCNQ1 in A7r5 cells (*P* < 0.05); 3 μM Lut significantly increased the expression of KCNQ1 compared with the NC group (*P* < 0.01); while 1 μM PDBu significantly inhibited the increasing KCNQ1 expression induced with Lut in A7r5 cells (*P* < 0.05). As shown in Figures 7C,D, immunofluorescence detection showed that 1 μM PDBu could significantly inhibit the expression of Kv7.1 protein in A7r5 cells compared with the control group, (*P* < 0.05); however, 3 μM Lut significantly increased the expression of Kv7.1 protein compared with the NC group (*P* < 0.05), while, the enhancement of Kv7.1 expression induced by 3 μM Lut was significatly reduced by 1 μM PDBu (*P* < 0.05).

As shown in Figure 7B, the expression of KCNQ1 in HG (high glucose) group was significantly decreased compared with NC group (*P* < 0.01); and compared with HG group, the expression of KCNQ1 in A7r5 cells was significantly decreased in HG + 1 μM PDBu group (*P* < 0.05). Compared with HG group, the expression of KCNQ1 in HG + 3 μM Lut group significantly increased (*P* < 0.05), but the enhancement could be significantly inhibited with 1 μM PDBu was added (*P* < 0.05). As shown in Figures 7E,F, the results of immunofluorescence detection showed that the fluorescence intensity of Kv7.1 protein in HG group was significantly reduced compared with the NC group (*P* < 0.01), and compared with HG group, the fluorescence intensity of Kv7.1 protein in A7r5 cells was significantly weakened in HG + 1 μM PDBu group (*P* < 0.05). Compared with HG group, the fluorescence intensity of Kv7.1 in HG + 3 μM Lut group was significantly enhanced (*P* < 0.05), however, addition of 1 μM PDBu significantly inhibited the enhancement of Kv7.1 fluorescence intensity compared with HG + 3 μM Lut group in A7r5 cells (*P* < 0.05).

## 3 Discussion

In this study, we observed that diabetes could damage blood vessels and cause vasomotor dysfunction, such as the enhancement of thoracic aorta vasoconstrictor function or the weakening of diastolic function. Luteolin can significantly reduce the contractile response of thoracic aorta in diabetic rats induced with 0.3 μM U46619 or 10 μM PE, and improve the vasodilation response of thoracic aorta in diabetic rats. As we know, the thoracic aorta consists of three layers: intima, media and outer membrane. The media is mainly composed of smooth muscle cells, which are the main factors regulating the contraction and relaxation function of the thoracic aorta. The sarcoplasmic reticulum of thoracic aorta smooth muscle cells is relatively undeveloped, and its contraction mainly depends on the inflow of outer calcium during cell depolarization. Potassium channels are important ion channels regulating the contraction and relaxation of arterial smooth muscle cells (Sahinturk et al., 2022). Kv7 channels, an important subclass of Kv channels, are active at more negative membrane potential than other Kv channels, making them perfect candidates for K^+^ currents that control the resting membrane potential of vascular smooth muscle cells (Zhong et al., 2010). The Kv7 channel consists of five subtypes Kv7.1-Kv7.5, encoded by the five known members of the KCNQ gene family, KCNQ1-KCNQ5 (Baldwin et al., 2020). Experiments have confirmed the presence of the Kv7 channel on vascular smooth muscle cells (Greenwood and Ohya, 2009). So we detected the expression of KCNQ1-KCNQ5 genes in the smooth muscle of thoracic aorta of normal rats, and found that the expression of KCNQ1, KCNQ4 and KCNQ5 genes in the thoracic aorta of rats was relatively high, which was consistent with the expression of human arterial smooth muscle found by Ng FL (Mani and Byron, 2011). Importantly, in rodent and human blood vessels, the Kv7.1 channel subtype plays a major role in regulating vascular smooth muscle membrane potential and contractility. In smooth muscle cells, when the expression of Kv7.1 channels is increased or activated, the outflow of potassium ions increases, which can hyperpolarize the cell membrane, reduce the opening of voltage-gated calcium channels, reduces the influx of calcium ions, and cause vasodilation. Therefore, in this experiment, we focused on the Kv7.1 channel.

Lut improved the function of blood vessels in diabetes may not solely stem from its hypoglycemic properties but are more likely mediated by the upregulation of Kv7.1 channels. Previous studies have shown that luteolin exerts vasodilatory effects through endothelial-dependent mechanisms involving NO or Ca^2+^ pathways (Roberts et al., 2013; Kamkaew et al., 2019; Qian et al., 2010). Our findings extend these observations by demonstrating that in diabetic conditions, luteolin can also activate Kv7.1 channels independent of endothelial function, highlighting its role in smooth muscle electrophysiological regulation. Compared to currently available antidiabetic agents such as SGLT2 inhibitors or GLP-1 receptor agonists, Lut not only reduces the blood glucose of diabetic rats, but also shows a direct protective effect on blood vessels, suggesting its unique potential as a vasoprotective therapeutic candidate. In experiment, the expression of KCNQ1 mRNA in thoracic aorta of diabetic rats was detected. The results show that KCNQ1 was decreased compared with the normal control group, which indicates that diabetes can damage the KCNQ1 of blood vessels. Meanwhile, the systolic and diastolic functions of the thoracic aortic vessels in diabetic rats were impaired, which might be related to the expression of KCNQ1. While Lut could significantly increase the expression of KCNQ1 in thoracic aorta of diabetic rats. Next we examined the effect of luteolin on the expression of KCNQ1 in A7r5 thoracic aorta smooth muscle cells under high glucose conditions. The results were consistent with the diabetic rat model, Lut can improve the expression of KCNQ1 under high glucose conditions. We have reached a preliminary conclusion KCNQ1 may be one of the main factors for Lut to improve the vasomotor dysfunction of thoracic aorta in diabetic rats. Kv7.1 may become a potential therapeutic target for delaying vascular complications of diabetes. This is meaningful for the clinical treatment of diabetes.

A large number of experiments have shown that the kinetic characteristics of transient outgoing potassium current are regulated by phosphorylation. Vasoconstrictor can inhibit K^+^ current by regulating Kv channel through PKC, causing contraction of vascular smooth muscle (Rainbow et al., 2009; Cheng et al., 2007). Activation of PKC also significantly inhibits Kv4.2 or Kv4.3 on frog egg cells (Birnbaum et al., 2004; Andersen et al., 2018). We want to know whether PKC regulates the expression of Kv7.1. We investigated whether PKC agonist mediated Lut to enhance the expression of Kv7.1 under high glucose conditions, and found that the expression of Kv7.1 under Lut to improve high glucose conditions was regulated by PKC agonist PDBu.

This experiment still has many deficiencies, for instance, some studies have mentioned that KCNE, together with calmodulin (CaM), may influence KCNQ channel function (Ciampa et al., 2011). However, due to time limitations, we were not able to include these experiments in the current study. We plan to investigate this in future work. In addition, considering that Lut may also directly act on the Kv7.1 channel, molecular docking simulations were performed, which indicated that Lut may bind to Kv7.1 with a predicted binding free energy of −9.5 kcal/mol. While this result does not provide direct experimental evidence of binding, it supports the possibility of a biologically relevant interaction between luteolin and Kv7.1 channels. Such an interaction may contribute to the enhancement of Kv currents. Interestingly, our earlier experimental data also support this possibility (Li et al., 2019). We plan to further explore this mechanism in our future studies.

In recent years, various drugs have been used in combination with hypoglycemic drugs such as metformin to prevent cardiovascular events. For instance, statins are used to lower LDL to prevent recurrence of cardiovascular events. However, the latter increases the risk of new-onset diabetes (Hori et al., 2019) Another research report suggests that drugs that are widely used, such as beta-blockers seem to have more or less influence on blood glucose levels (Tsujimoto et al., 2017) It is an urgent need in clinical practice to find drugs that can be used in combination with hypoglycemic drugs to protect blood vessels and have few adverse reactions. The outstanding protective effect of luteolin on the blood vessels of diabetic rats makes it possible to be used as one of the combination drugs of hypoglycemic drugs to delay the occurrence and development of diabetic vascular complications. When Lut used in combination with hypoglycemic drugs such as metformin, SGLT2 inhibitors, and GLP-1 receptor agonists, may become an ideal clinical medication regimen for patients with diabetes complicated with cardiovascular complications, reducing the occurrence of cardiovascular events.

## 4 Materials and methods

### 4.1 Experimental animals and cells

Thirty-six male SPF Sprague-Dawley (SD) rats weighing (270–320 g, eight-week-old) were purchased from Sibeifu (Beijing) Biotechnology (license key: SCXK2024-0001), the rats were fed adaptively for a week, with temperature (22 °C–24 °C), humidity (50%), 12 h light/12 h dark cycles and free for food and water. All animal experiments in this study were approved by the Ethics Committee of Shanxi Medical University Fenyang College (2025004). And the three R principle of reduction, substitution and optimization were strictly followed. Rat thoracic aortic smooth muscle cells (A7r5 cells) were used in this study.

### 4.2 Reagents and instruments

Luteolin (Lut, HPLC ≥98%) (Macklin Biochemical Technology Co., Ltd., Shanghai, China); Streptozotocin (STZ), Acetylcholine (ACh), 9,11-dideoxy-9α,11α-methanoepoxy-Prostaglandin-F_2α_ (U46619), 4-aminopyridine (4-AP), 10,10-bis(4-pyridinylmethyl)-9(10H)-anthracenone (XE-991), N-(2,4,6-Trimethylphenyl)-bicyclo [2.2.1]Heptane-2-carboxamide (ML213), phenylephrine (PE), 5-hydroxytryptamine (5-HT), Chromanol 293B and phorbol 12,13-dibutyrate (PDBu) were purchased from Sigma-Aldrich (Shanghai) Trading Co., Ltd.; Sodium nitroprusside (SNP) was purchased from HS Pharmaceuticals Co., Ltd.; Barium chloride and citric acid were obtained from Tianjin Obo Kai Chemical Co., Ltd.; Trisodium citrate was obtained from Tianjin Fengchuan Chemical Reagent Technology Co., Ltd.; Sodium carboxymethylcellulose was obtained from Tianjin Beichen District Fangzheng Reagent; The primer of β-actin and KCNQ1 were obtained from Sangon Biotech (Shanghai) Co., Ltd.; Antifade mounting medium, DAPI Stain Solution, NaCl, KCl, CaCl_2_, MgCl_2_·6H_2_O, 4-(2-Hydroxyethyl)piperazine-1-ethanesulfonic acid (HEPES), D-glucose were purchased from Sangton Biotech (Shanghai) Co., Ltd.; TRIzol™ Reagent (Mei5 Bioservices Co.Ltd., MF034), Diethylpyrocarbonate Water (DEPC, R1600), Penicillin Streptomycin Amphotericin B Mixed Solution (P7630) were obtained from Beijing Solarbio Science & Technology Co., Ltd.; PrimeScript™ RT reagent Kit with gDNA Eraser (Perfect Real Time) (RR047A), TB Green^®^ Premix Ex Taq™ II (Tli RNaseH Plus) (RR820A) were obtained from Takara; Cell lysis buffer (P0013), protease inhibitor (PMSF) (ST506) were obtained from Beyotime Biotechnology; BCA protein concentration determination kit (AR0146), SDS-PAGE Protein loading buffer (denaturing) (5×) (P0015L), Supersensitive ECL chemiluminescence ready-to-use substrate (AR117I), LG DMEM were purchased from Wuhan Boster Biological Technology.,Ltd.; The KCNQ1 antibody (APC-022) used for cellular immunofluorescence was provided by Alomone Labs. Goat anti-rabbit IgG H&L (Alexa Fluor^®^ 488) (ab150077) which were pre-adsorbed secondary antibody was obtained from Aibo Kang (Shanghai) Technology Co., Ltd.; No serum non-programmed cell freeze (MA0405), 0.25% pancreatic enzyme digestion fluid (modified form Meilunbio) (MA0232) and PBS (1×) (MA0015) were purchased from Dalian Meilun Biotechnology Co., Ltd., cellmax superior fetal bovine serum (Saiaomei Cell Technology (Beijing) Co., Ltd.,SA211); TG assay kit (A110-1-1), TC assay kit (A111-1-1), LDL-C assay kit (A113-1-1), HDL-C assay kit (A112-1-1) were purchased from Nanjing Jiancheng Bioengineering Institute; HV-4 thermostatic perfusion system for isolated tissues and organs (Beijing Boyi Times Medical Technology Co., Ltd.); High-pressure sterilizer (Panasonic); High speed freezing centrifuge (Eppendorf); Ultramicro biodetector (NanoDrop); Ultrasonic cell disruptor (Omni International); ELIASA (Bio Tek); Vertical electrophoresis apparatus, transmembrane apparatus, Chemiluminescence imaging system, fluorescent quantitative PCR instrument and ordinary PCR instrument were obtained from Bio-Rad; Microscopez (Leica); Ice machine (Beijing Changliu Scientific Instrument Co., Ltd.).

### 4.3 Experimental methods

#### 4.3.1 Animal model and treatment (Srinivasan et al., 2005; Furman, 2021)

Thirty-six male SPF Sprague-Dawley rats were adaptively fed for 7 days, and then, fasting blood glucose (FBG) were measured with fasted for 12 h, rats with fasting blood glucose of 3.9–6.1 mM levels were considered as experimental animals. The rats were randomly divided into two groups of control group and high-fat feeding (HFD) group, the control group received normal feeding for 8 weeks, while the HFD group were fed with high-carbohydrate and high-fat feed (D12451, Mouse Youtai Biotechnology Co.Ltd.) for 8 weeks. Eight weeks later, the control group was randomly divided into two groups for control (n = 7) and control + Lut (n = 7). The HFD group were intraperitoneally injected with streptozotocin (STZ), which was dissolved in citric acid-sodium citrate buffer citrate, pH 4.5, at the dose of 30 mg/kg. The control group received an equal volume of citric acid-sodium citrate buffer, 3 days and 7 days later, FBG was measured by tail amputation, rats with fasting blood glucose greater than 11.1 mM are considered to be diabetic. The diabetic rats were randomly divided into two groups for DM (n = 8) group and DM + Lut (n = 8) group, a week later, the group of NC + Lut and DM + Lut were received Luteolin (80 mg/kg/d) by gavage for 12 weeks respectively, while the group of DM and NC were received the same volume and of 0.5% CMC. FBG and body weight were measured weekly. After 12 weeks, the animals were anesthesia with sodium pentobarbital (40 mg/kg), and the abdominal aorta were isolated first, blood was collected with disposable blood collection needle and vacuum negative pressure, and then the rat thoracic aorta was isolated, part of which was used for vascular ring experiments, and the remained part was frozen in −80 °C for qRT-PCR experiments.

#### 4.3.2 Cell culture and treatment

A7r5 cells were presented by Shanxi Medical University, the recorved A7r5 cells were inoculated in complete medium with 10% fetal bovine serum, Penicillin Streptomycin Amphotericin B Mixed Solution and DMEM with low glucose under 37 °C, 5% CO_2_ condition. After the cells reached 80% confluence, the cells were digested by pancreatic enzyme and then seeded in a 6-well culture plate, after 24 h of normal treatment, and then 12 h of serum-free treatment, the cells were cultured for 24 h according to the following groups: (1) Normal Lut gradient experiment: NC (5.5 mM Glucose, Normal Glucose), NC + 1 μM Lut, NC + 3 μM Lut, NC + 10 μM Lut, NC + 30 μM Lut, NC + 100 μM Lut; (2)Normal Kv7.1 inhibitor test: NC, NC + 1 μM chromanol 293B, NC + 3 μM Lut, NC + 1 μM chromanol 293B+ 3 μM Lut; (3) HG (High Glucose) Lut gradient experiment: NC, HG (30 mM Glucose), HG + 1 μM Lut, HG + 3 μM Lut, HG + 10 μM Lut, HG + 30 μM Lut, HG + 100 μM Lut; (4) High glucose Kv7.1 inhibitor experiments: HG group, HG + 1 μM chromanol 293B, HG + 3 μM Lut, HG +1 μM chromanol 293B+ 3 μM Lut; (5) Normal PKC agonist experiment: NC, NC + 1 μM PDBu, NC + 3 μM Lut、NC + 1 μM PDBu +3 μM Lut; (6) HG PKC agonist experiment: NC, HG, HG + 1 μM PDBu, HG + 3 μM Lut, HG + 1 μM PDBu +3 μM Lut. Cells were collected after 24 h of culture. Preparation of low-glucose (5.5 mM Glucose) complete medium: 10% fetal bovine serum (5 mL) + 1% Penicillin Streptomycin Amphotericin B Mixed Solution (0.5 mL) + low-glucose DMEM (44.5 mL), Preparation of high-glucose (30 mM Glucose) complete medium: 10% fetal bovine serum (5 mL) + 1% Penicillin Streptomycin Amphotericin B Mixed Solution (0.5 mL) + low-glucose DMEM (44.5 mL) + 0.23 g glucose powder, the bacteria were removed by 0.22 μm disposable needle filter. The solution configuration takes 50 mL medium as an example.

#### 4.3.3 Biochemical studies

The blood of each group of rats was collected, and then centrifuged at 3000 r for 15 min, and then the serum was collected. TC was tested with a TC assay kit, TG was measured with a TG assay kit, HDL-C was determined with an HDL-C assay kit, and LDL-C was measured with an LDL-C assay kit.

#### 4.3.4 Vascular ring tension was measured by myotonometer (Niu et al., 2008)

1. After the rats in each group were raised for the designated period, they were anesthetized with sodium pentobarbital (40 mg/kg) by intraperitoneally injected, isolate the thoracic aorta and put into pre-cooled oxygen-saturated 4 °C normal HEPES solution, and then use ophthalmic scissors to separate the tissue around the blood vessel along the tube wall until the tissue separates clean, cutting the separated blood vessels into 1.5 cm segments. Remove vascular endothelium mechanically: use eye tweezers to wrap cotton wool and gently rub the vascular endothelium two or three times to remove the vascular endothelium. The prepared vascular ring of the thoracic aorta was suspended in a tonometer bath containing 10 mL normal HEPES solution. Oxygen was continuously injected, pH = 7.4, stable at 37 °C for 1 h, and then adjust the base tension to 1.5 g, rebalance and stabilize for 1 h, and replace the HEPES solution every 15 min in the bath. The vascular ring was stimulated twice with 60 mM KCl, if the amplitude difference between the two vasoconstrictions is not more than 10%, it is considered that the vascular ring response is good, and subsequent experiments can be carried out. The vascular rings were stimulated with 60 mM KCl at the end of the experiment. It was considered that the reactivity and stability of the vascular rings were good if the amplitude of vascular contraction was no more than 10% different from that before the experiment, and the experimental results were considered reliable and credible.
2. Examination for vascular endothelial removal: the thoracic aortic ring was pre-constricted with 1 μM PE, and when it reached the plateau, the thoracic aortic ring was dilated with 10 μM ACh. If the diastolic percentage is less than 10%, the vascular endothelium is considered to be removed. The percentage of vasodilation is equal to ACh dilation divided by PE precontraction.
3. The normal HEPES was composed of (mM): 144 NaCl, 5.8 KCl, 1.2 MgCl_2_·6H_2_O, 2.5 CaCl_2_, 11.0 D-glucose, 5 HEPES, PH = 7.40. The 60 mM KCl was composed of (mM): 89.8 NaCl, 60 KCl, 1.2 MgCl_2_·6H_2_O, 2.5 CaCl_2_, 11.0 D-glucose, 5 Hepes, PH = 7.40.
4. The constriction response of vascular ring was detected: the vascular ring was stimulated with 60 mM KCl, 0.3 μM U46619, 10 μM PE and 10 mM 4-AP, respectively. The contractile effect of 60 mM KCl, 0.3 μM U46619, 10 μM PE and 10 mM 4-AP on thoracic aorta was observed.
5. The diastolic response of vascular ring was measured: the vascular ring was pre-constricted with 10 μM PE or 0.3 μM U46619, respectively. When the constriction reached the plateau, ACh or SNP was added to observe the vasodilation response in each group.

#### 4.3.5 Real-time fluorescence quantitative PCR was used to detect KCNQ1

Total RNA extraction of thoracic aorta tissue and A7r5 cells (Yu et al., 2022): (1) the thoracic aorta tissues were collected from each group of rats, and then the tissues were firstly cut up with ophthalmic scissors, then pour in liquid nitrogen and grind with a mortar. Then ground tissue was collected and 1 mL Trizol was added. In the cell experiment, 1 mL Trizol was added after the cells were collected, and the solution was repeatedly blown and mixed until the solution was viscous. Then standing for 5 min, centrifuged at 13000 r/min, 4 °C for 2 min (2) the supernatant was collected, and then, added 200 μL of chloroform, stand for 10 min, and then centrifuged at 13000 r, 4 °C for 15 min (3) take the supernatant, add an equal amount of isopropyl alcohol, stand at room temperature for 10 min, centrifuge at 13000 r/min, 4 °C for 10 min (4) discard the supernatant, add 1 mL 75% ethanol, 13000 r/min, centrifuge at 4 °C for 3 min (5) discard the supernatant, add 1 mL 75% ethanol, 13000 r/min, reverse centrifuge at 4 °C for 3 min (6) discard the supernatant, add 1 mL 75% ethanol, 13000 r/min, and centrifuge at 4 °C for 3 min (7) discard the supernatant, dry the RNA and precipitate it until translucent, add 20 μL enzyme-free sterile water, and then determine the purity and concentration of the RNA. The primer sequences used in this study are presented in Table 1.

**TABLE 1:** Primers sequences for qRT-PCR.

Gene	Sequence (5’→3′)
β-ACTIN	F: TGT​CAC​CAA​CTG​GGA​CGA​TA
	R: GGG​GTG​TTG​AAG​GTC​TCA​AA
KCNQ1 (Kv channel subfamily KQT member 1)	F: TGG​GTT​CCA​AAG​GGC​AAG​TGT​TC
	R: GCT​CCT​GGC​GGT​GAA​TGA​AGA​C

#### 4.3.6 Kv7.1 protein was detected by cellular immunofluorescence staining (Xu et al., 2024)

(1) 24-well plate cell climbing tablets, after the cells were attached to the wall, the medium was discarded and washed with PBS three times for 5 min each time; (2) Fixed with paraformaldehyde for 20 min, and then washed with PBS three times for 5 min each time; (3) Closed with 10% goat serum at room temperature for 1 h, added primary antibody, refrigerated at 4 °C overnight, and washed with PBS three times the next day; (4) Add fluorescent secondary antibody, incubate at room temperature for 1 h, avoid light, rinse with PBS three times, stain the nucleus with DAPI for 2 min, remove the cell slide, add anti-fluorescent quench agent, seal the film, observe with fluorescence microscope.

### 4.4 Statistical analysis

All statistical analysis were performed using the SPSS 22.0 software or GraphPad Prism 6 (GraphPad software version 6.0) software. All data were expressed as means ± standard deviation. Normal distribution test was performed on the data first. When the data met the normal distribution, homogeneity of variance and one-way ANOVA were performed. T-test was used to analyze the difference in inhibition experiments. The value of *n* refers to the number of rats or A7r5 cells. *P* < 0.05 were considered to indicate significance.

## Data Availability

The original contributions presented in the study are included in the article/supplementary material, further inquiries can be directed to the corresponding authors.
